# Changes in Home-visit Care Utilization and Outcomes During the Coronavirus Disease 2019 Pandemic: Comparison Between Patients With and Without Cancer

**DOI:** 10.2188/jea.JE20250543

**Published:** 2026-10-05

**Authors:** Yu Sun, Jun Komiyama, Atsushi Miyawaki, Masao Iwagami, Nobuo Sakata, Satoru Yoshie, Tomoko Ito, Kimikazu Kashiwagi, Nanako Tamiya

**Affiliations:** 1 Department of Health Services Research, Institute of Medicine, University of Tsukuba, Ibaraki, Japan; 2 Health Services Research and Development Center, University of Tsukuba, Ibaraki, Japan; 3 Department of Primary Care and Medical Education, Institute of Medicine, University of Tsukuba, Ibaraki, Japan; 4 Department of Public Health and Health Policy, Institute of Medicine, University of Tsukuba, Ibaraki, Japan; 5 Department of Digital Health, Institute of Medicine, University of Tsukuba, Ibaraki, Japan; 6 Heisei Medical Welfare Group Research Institute, Tokyo, Japan; 7 Department of Home Healthcare, Setagaya Memorial Hospital, Tokyo, Japan; 8 Institute of Gerontology, University of Tokyo, Tokyo, Japan; 9 Institute for Future Initiatives, University of Tokyo, Tokyo, Japan; 10 Department of Health Policy and Management, School of Medicine, Keio University, Tokyo, Japan; 11 School of Medicine, Hiroshima University, Hiroshima, Japan; 12 Faculty of Nursing, National College of Nursing, Tokyo, Japan

**Keywords:** home health care, end-of-life care, coronavirus disease 2019, pandemic, home care support clinics/hospitals, cancer

## Abstract

**Background:**

The coronavirus disease 2019 (COVID-19) pandemic disrupted access to healthcare for homebound older adults, particularly those with cancer. We compared changes in the initiation and outcomes of physician home-visit care among older Japanese patients with and without cancer during the pandemic.

**Methods:**

We conducted a retrospective observational study using national medical claims data of patients aged ≥65 years who began physician home visits between April 2018 and December 2020 in Japan. The proportions of all-cause death, in-home death, and at least one hospitalization within 90 days after the initiation of home-visit care were compared between the pre-pandemic (April 2018 to December 2019) and pandemic periods (January 2020 to December 2020), stratified by cancer diagnosis. Differences between the cancer and non-cancer groups were estimated using linear regression models with an interaction term for cancer status and pandemic period.

**Results:**

Among 509,718 patients (251,311 with cancer), the initiation of home-visit care increased in both groups during the pandemic. All-cause and in-home death rates increased and hospitalization rates decreased in both groups, with greater changes among patients with cancer: 1.6% (95% confidence interval [CI], 1.1–2.1) for all-cause death, 3.8% (95% CI, 3.4–4.3) for in-home death, and −2.9% (95% CI, −3.7 to −2.2) for hospitalization.

**Conclusion:**

During the COVID-19 pandemic, physician home-visit care expanded, and patients with cancer showed a greater shift toward home-based end-of-life care. These findings highlight the need for targeted resource allocation strategies to support home-visit physicians caring for patients with cancer during public health emergencies.

## INTRODUCTION

The coronavirus disease 2019 (COVID-19) pandemic markedly disrupted healthcare delivery and altered patterns of service use. Hospital bed shortages limited access to inpatient treatment when patients’ conditions worsened, and fear of infection further discouraged hospital admission.^1^^–^^3^ As a result, demand for home-based healthcare and the number of deaths occurring at home increased in both Japan and other countries.^4^^–^^6^

In Japan, physician regular home-visit care plays a central role in supporting home-based healthcare for older adults. Among these patients, those with cancer represent a distinct clinical population, as home-visit care is often initiated with the primary goal of enabling end-of-life care at home.^7^ In contrast, patients without cancer typically receive home-visit care for long-term management of slowly progressive conditions, such as frailty or dementia, with a focus on maintaining daily functioning. Therefore, the clinical purpose and intensity of home-visit care differ substantially between the two groups, and the COVID-19 pandemic may have affected their care needs in different ways.

Previous studies have documented increased home deaths during the pandemic in Japan, particularly among patients with cancer.^6^^,^^8^^,^^9^ However, these reports include deaths among individuals who were not receiving physician home-visit care before death, as well as deaths occurring in residential facilities, such as group homes for older individuals with dementia, home hospice, and older adult housing equipped with care services (*Sabisutsuki koreisya muke Jutaku*). Therefore, whether home deaths among patients with cancer were supported by physician home-visit care prior to death, or how the pandemic altered the initiation of such care was unclear from these studies. Our previous study showed an increase in newly initiated home-visit care and early home deaths among patients with cancer during the pandemic; however, the findings were limited to a small number of facilities in one prefecture.^10^ To date, no nationwide study has examined how the initiation and short-term outcomes of physician home-visit care have changed for patients with versus without cancer.

To address this knowledge gap, we compared the utilization and short-term outcomes of newly initiated physician regular home-visit care among older patients with and without cancer before and during the COVID-19 pandemic in Japan. In this study, we aimed to provide a more nuanced understanding of how the pandemic has affected home healthcare in Japan, particularly from the perspective of cancer care. Our findings can help clinicians anticipate patients who may require timely home-visit care during any possible future pandemic and proactively prepare care systems to meet these needs. They can also assist policymakers in developing contingency strategies—such as flexible staffing support, expanded telemedicine models, and strengthened regional collaboration—to ensure that home-visit and palliative care resources are allocated efficiently to those with the highest needs.

## METHODS

### Home healthcare in Japan

In Japan, home healthcare refers to physicians providing regular home visits, which are reimbursed under a health insurance scheme.^11^^,^^12^ Physician regular home-visit care is initiated for patients unable to attend outpatient clinics. Prior to the initiation of home-visit care, preliminary interviews are typically conducted to gather patient information and assess their care needs. Once initiated, physicians are required to make regular home visits, typically once or twice per month, depending on the patient’s medical condition. According to the Ministry of Health, Labour and Welfare (MHLW), physician home-visit care serves four primary functions: facilitating discharge assistance through collaboration with hospital staff, providing daily life support that includes family caregiver assistance and palliative care, offering emergency home visits for acute illnesses or exacerbations of chronic conditions, and delivering end-of-life care at home.^12^

### Data source and study participants

This study retrospectively analyzed data from the National Database of Health Insurance Claims (NDB) between April 2012 and March 2021. The NDB is a Japanese administrative claims database provided by the MHLW that covers approximately 98% of the data on healthcare services provided by healthcare institutions.^13^ It contains claims information, including anonymous individual identification numbers, age, sex, disease codes, medical care procedures, and drug prescriptions. Procedures and medications are coded using the original Japanese codes. Diagnoses can be linked to the International Classification of Diseases, 10th Revision (ICD-10) codes.

The study included patients aged ≥65 years who initiated physician regular home visits between April 2018 and December 2020. The washout period was from April 2012 to March 2018, during which patients who had received physician regular home-visit care were excluded from the analysis. Physician regular home-visit care was identified based on the combination of claims for the “Physician Home Visit Fee (*zaitaku kanja hōmon shinryōryō*)” and either the “Home Medical Care Management Fee (*zaitaku ji igaku sōgō kanri-ryō*)” or the “Home Cancer Care Management Fee (*zaitaku gan igaku sōgō kanri-ryō*).”

### Patient characteristics

Patient characteristics at the initiation of regular home-visit care included age (65–74, 75–84, 85–94, ≥95 years), sex, and medical diagnosis at the start of regular home visits (cerebrovascular diseases, cardiac diseases, lower respiratory tract diseases, joint diseases, dementia, fractures, and cancer). Medical diagnoses were identified during the 3 months before the start of the regular home visit, and the “suspected” diagnosis codes were excluded. eMaterial 1 provides the corresponding ICD-10 codes.

### Outcomes

As outcomes of interest, we assessed the utilization of several medical services and clinical outcomes within 90 days of the initiation of home-visit care. These included (i) all-cause death, (ii) in-home death, and (iii) at least one hospitalization. Additionally, we evaluated (iv) at least one emergency home visit and (v) at least one telephone follow-up consultation. All-cause death was confirmed by the claims-based definition (defined as patients for whom the discharge/disease status recorded in the most recent claims was death) or by surcharges for the death certificate or end-of-life care at home. A previous validation study on the accuracy of death information in claims data showed that the sensitivity and specificity of inpatient death were 94.3% and 98.5%, respectively.^14^ In-home death was defined by surcharges for the death certificate or end-of-life care at home.

### Statistical analysis

First, we described the characteristics of the study population and plotted the monthly trends in the number of patients who newly initiated physician regular home-visit care, stratified based on cancer diagnosis.

Second, we illustrated the monthly trends in the proportion of outcomes within 90 days of initiating home-visit care.

Third, to examine how the changes in the five outcomes before and after the COVID-19 pandemic varied between patients with and without cancer, we performed linear regression analyses that regressed each outcome on the following three variables: (1) indicator of the status of patients with cancer, (2) indicator of the pandemic period, and (3) their interaction term. We reported the coefficient of the interaction term, which captures the relative change in percentage points in each outcome in patients with cancer compared with those without cancer from the pre-pandemic to the pandemic period. We adjusted for prefecture fixed effects and patient covariates (age category, sex, and medical diagnosis at the start of regular home visits) to account for regional heterogeneity and case mix. Standard errors were clustered at the prefecture level to account for correlations between patients within the same prefecture. Given that the first confirmed case of COVID-19 in Japan was identified in January 2020 and the first state of emergency related to COVID-19 was declared on April 7, 2020, in Japan,^15^ we considered that the 90-day outcomes might differ for patients who started home-visit care after January 2020. Therefore, we defined the pre-pandemic period as patients who began physician regular home-visit care from April 2018 to December 2019 and the pandemic period as those who initiated care from January 2020 to December 2020. In the regression, the pandemic period indicator was coded 0 for the pre-pandemic period and 1 for the pandemic period.

For sensitivity analysis, we redefined the boundary between the pre-pandemic and pandemic periods as April 2020, instead of January 2020 as used in the main analysis, and repeated the same analytical procedures.

As additional analyses, to identify subgroups with greater changes in new initiation of home-visit care and outcomes within 90 days between patients with and without cancer, we performed stratified analyses by three variables: (1) age (<85 vs ≥85 years), (2) sex, and (3) region (prefectures initially subject to the state of emergency [Tokyo, Kanagawa, Saitama, Chiba, Osaka, Hyogo, and Fukuoka] vs all other prefectures). Moreover, we conducted cancer-type-specific analyses by sex, focusing on major cancers with high incidence and mortality in Japan (male patients: colorectal, lung, gastric, pancreatic, and prostate cancer; female patients: colorectal, lung, gastric, pancreatic, and breast cancer). For each analysis, we examined the monthly number of patients who newly initiated physician regular home-visit care and their 90-day outcomes in the cancer and non-cancer groups.

All statistical analyses were conducted using STATA version 18 (StataCorp, College Station, TX, USA). Two-sided *P* values <0.05 were considered statistically significant.

### Ethical considerations

This study was approved by the Ethics Committee of the University of Tsukuba (approval number 1729-1). The need for informed consent was waived because the NDB data were anonymized before they were made available to the researchers.

## RESULTS

### Patient characteristics and monthly trends in newly initiated home-visit care

In total, 509,718 patients were included in the analysis, of whom 251,311 had cancer. Table 1 shows the characteristics of the study population. The most common age group was 85–94 years, with 53.5% of the patients being female. Among the diagnoses, cardiac, joint, and lower respiratory tract diseases were the most prevalent. Figure 1 shows the monthly trends in the number of patients who newly initiated physician regular home-visit care, stratified based on cancer diagnosis. The number of patients with and without cancer who initiated regular home visits increased after the onset of the COVID-19 pandemic.

**Figure 1.**
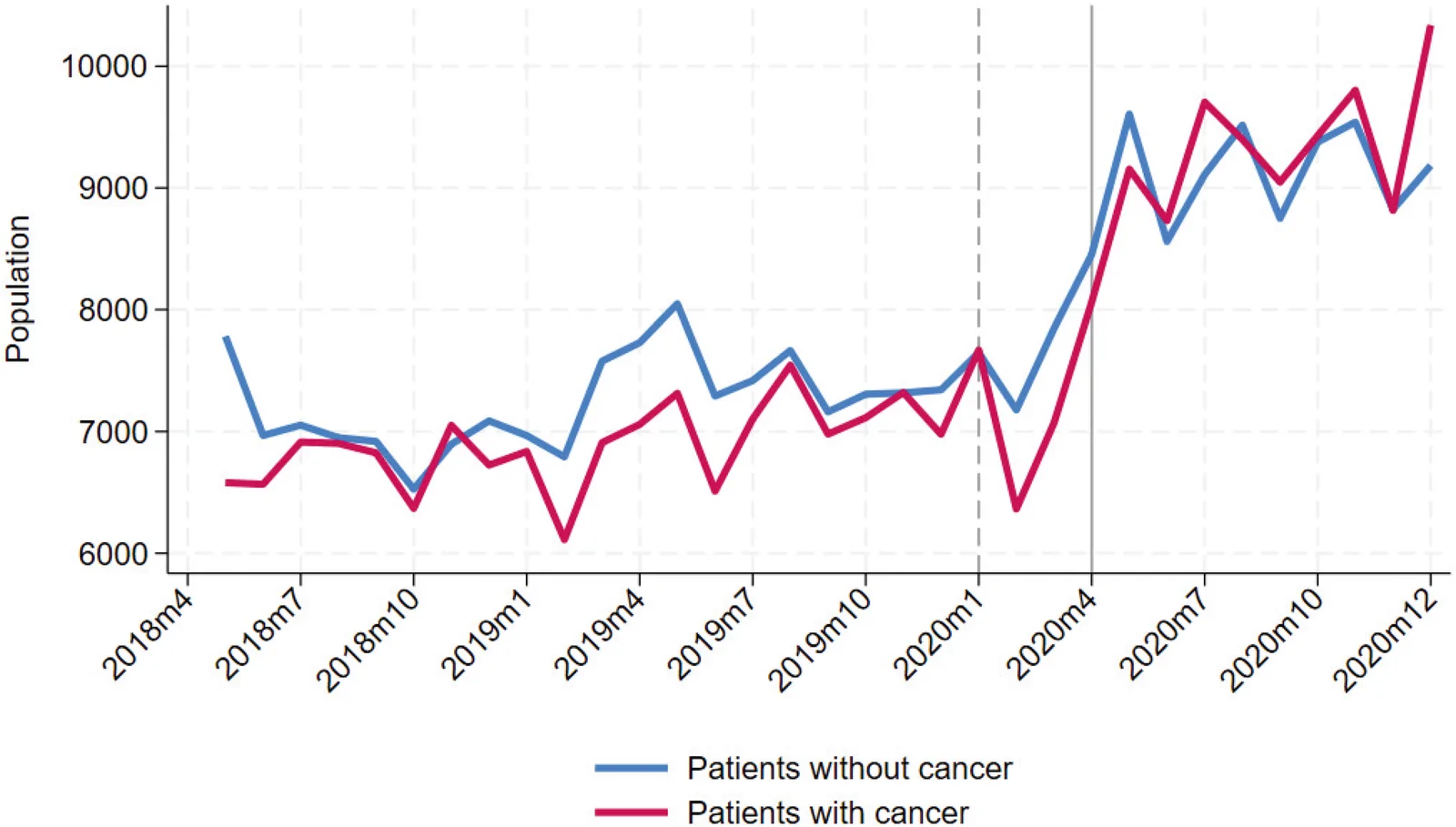
Monthly trends in the number of patients who newly initiated physician regular home visit, stratified by cancer diagnosis. The gray dashed line indicates the month of the first confirmed coronavirus disease 2019 case in Japan and marks the boundary between the pre-pandemic and pandemic periods defined in this study. The gray solid line represents the month in which a state of emergency was declared in Japan.

**Table 1.** Characteristics of the study population

	All	Pre-pandemic period^*^	Pandemic period^*^

	*n* = 509,718	*n* = 297,843	*n* = 211,875

	*n* (%)	*n* (%)	*n* (%)
**Age category**			
65–74 years	81,495 (16.0)	47,263 (15.9)	34,132 (16.1)
75–84 years	180,037 (35.3)	105,982 (35.6)	74,055 (35.0)
85–94 years	208,280 (40.9)	121,398 (40.8)	86,882 (41.0)
≥95 years	40,006 (7.8)	23,200 (7.8)	16,806 (7.9)
**Sex: female**	272,514 (53.5)	159,351 (53.5)	113,163 (53.4)
**Disease**			
Cerebrovascular diseases	195,437 (38.3)	116,260 (39.0)	79,177 (37.4)
Cardiac diseases	361,775 (71.0)	209,905 (70.5)	151,870 (71.7)
Lower respiratory tract disease	285,179 (55.9)	167,574 (56.3)	117,605 (55.5)
Joint diseases	339,670 (66.6)	197,731 (66.4)	141,939 (67.0)
Dementia	181,548 (35.6)	106,690 (35.8)	74,858 (35.3)
Fractures	104,422 (20.5)	60,966 (20.5)	43,456 (20.5)
Cancer	251,311 (49.3)	145,386 (48.8)	105,925 (50.0)

^*^The pre-pandemic period included patients who newly initiated physician regular home-visit care between April 2018 and December 2019, while the pandemic period included those who initiated care between January 2020 and December 2020.

### Monthly trends in the proportion of outcomes

Figure 2 shows the monthly trends in the five 90-day outcomes among patients who initiated regular home visits each month, stratified based on cancer diagnosis. Table 2 summarizes the outcomes. During the pandemic, the proportions of all-cause death and in-home death increased and hospitalizations decreased in both groups, with greater changes among patients with cancer (all-cause death, 44.9–47.2%; in-home death, 27.5–32.5%; hospitalization, 37.4–32.6%) than among patients without cancer (11.2–11.7%, 6.8–7.7%, and 21.0–18.9%, respectively). Emergency home visits increased only among patients with cancer, whereas telephone follow-up consultations increased in both groups.

**Figure 2.**
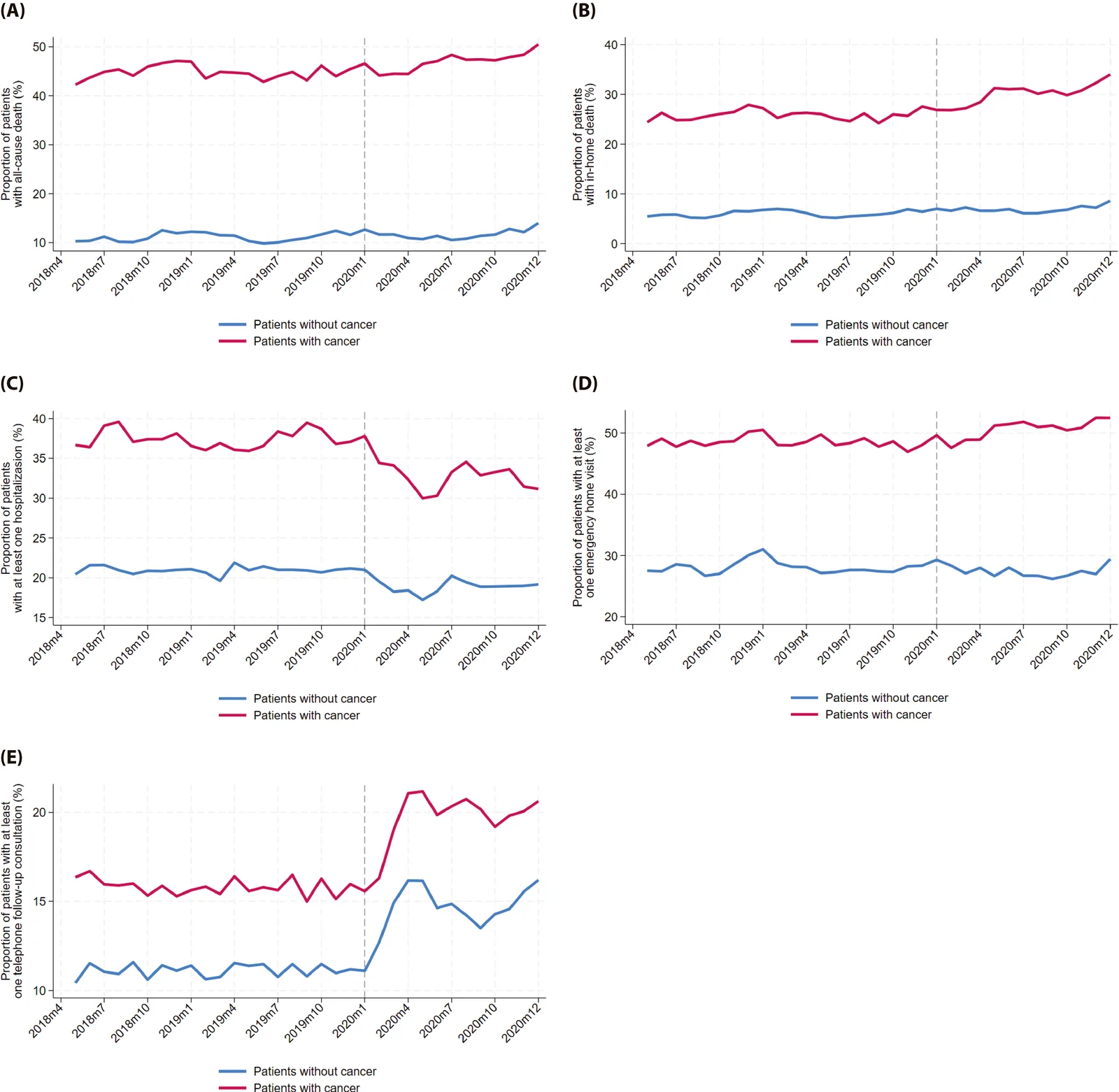
Monthly trends in 90-day outcomes among patients who initiated physician regular home-visit each month, stratified by cancer diagnosis. (**A**) all-death; (**B**) in-home death; (**C**) at least one hospitalization; (**D**) at least one emergency home visit; (**E**) at least one telephone follow-up consultation. The gray dashed line indicates the month of the first confirmed coronavirus disease 2019 case in Japan and marks the boundary between the pre-pandemic and pandemic periods defined in this study.

**Table 2.** 90-day outcomes among patients who newly initiated physician home-visit care, stratified by pandemic period and cancer diagnosis

	Pre-pandemic period^*^			Pandemic period^*^		

	Total patients	Patients with cancer	Patients without cancer	Total patients	Patients with cancer	Patients without cancer

	*n* = 297,843	*n* = 145,386	*n* = 152,457	*n* = 211,875	*n* = 105,925	*n* = 105,950

	(%)	*n* (%)	*n* (%)	*n* (%)	*n* (%)	*n* (%)
(i) All-cause deaths	82,314 (27.6)	65,247 (44.9)	17,067 (11.2)	62,340 (29.4)	49,988 (47.2)	12,352 (11.7)
(ii) In-home deaths	50,352 (16.9)	40,038 (27.5)	10,314 (6.8)	42,541 (20.1)	34,402 (32.5)	8,139 (7.7)
(iii) At least one hospitalization	86,377 (29.0)	54,428 (37.4)	31,949 (21.0)	54,452 (25.7)	34,480 (32.6)	19,972 (18.9)
(iv) At least one emergency home visit	113,495 (38.1)	70,634 (48.6)	42,861 (28.1)	82,816 (39.1)	53,851 (50.8)	28,965 (27.3)
(v) At least one telephone follow-up consultation	39,945 (13.4)	22,986 (15.8)	16,959 (11.1)	36,889 (17.4)	21,158 (20.0)	15,731 (14.8)

^*^The pre-pandemic period included patients who newly initiated physician regular home-visit care between April 2018 and December 2019, while the pandemic period included those who initiated care between January 2020 and December 2020.

### Relative change in outcomes in patients with cancer compared with those without cancer

Table 3 shows the result of the multivariable regression analyses. The increases in all-cause death and in-home deaths before and after the onset of the pandemic were greater among patients with cancer compared with those without cancer, by 1.6 percentage points (pp) (95% confidence interval [CI], 1.1–2.1 pp) and 3.8 pp (95% CI, 3.4–4.3 pp), respectively. We also found that the decrease in hospitalization was greater among patients with cancer compared with those without cancer, by −2.9 pp (95% CI, −3.7 to −2.2 pp). Increases in both emergency home visits and telephone follow-up consultations were significantly higher in the cancer group than in the non-cancer group, by 2.8 pp (95% CI, 2.2–3.5 pp) and 0.5 pp (95% CI, 0.0–0.9 pp), respectively.

**Table 3.** Results of linear regression analyses examining changes in 90-day outcomes before and during the COVID-19 pandemic among patients with and without cancer

	Cancer diagnosis					

	Patients with cancer		Patients without cancer			

	Adjusted difference (95% CI), percentage points	*P* value	Adjusted difference (95% CI), percentage points	*P* value	Relative difference (95% CI), percentage points	*P* value
(i) All-cause deaths	2.1 (1.4–2.8)	<0.001	0.5 (0.2–0.9)	0.002	1.6 (1.1–2.1)	<0.001
(ii) In-home deaths	4.8 (4.2–5.3)	<0.001	1.0 (0.7–1.2)	<0.001	3.8 (3.4–4.3)	<0.001
(iii) At least one hospitalization	−4.9 (−5.7 to −4.0)	<0.001	−2.0 (−2.4 to −1.6)	<0.001	−2.9 (−3.7 to −2.2)	<0.001
(iv) At least one emergency home visit	2.1 (1.4–2.9)	<0.001	−0.7 (−1.2 to −0.2)	<0.001	2.8 (2.2–3.5)	<0.001
(v) At least one telephone follow-up consultation	4.1 (3.5–4.8)	<0.001	3.7 (3.1–4.2)	<0.001	0.5 (0.0–0.9)	0.042

CI, confidence interval; COVID-19, coronavirus disease 2019.

Linear regression analyses were performed by regressing each outcome on three variables: (1) a cancer indicator, (2) a pandemic-period indicator, and (3) their interaction. Models were adjusted for prefecture fixed effects and patient covariates (age category, sex, and medical diagnosis at the start of regular home visits) to account for regional heterogeneity and case-mix. Standard errors were clustered at the prefecture level to account for correlations among patients within the same prefecture.

### Sensitivity analyses

The results of redefining April 2020 as the beginning of the pandemic period were consistent with the main analysis, and the differences in the five outcomes between patients with and without cancer became slightly more pronounced than in the primary analysis (eTable 1 and eTable 2).

### Subgroup analyses

In age-stratified analyses, patients aged <85 years were more likely to have cancer, whereas those aged ≥85 years were more likely to be patients without cancer in both periods. The number of patients initiating home-visit care increased after the pandemic onset in both age groups; however, this increase was particularly pronounced among patients with cancer aged <85 years. Regarding outcomes, both age groups showed trends consistent with the main analysis: greater increases in all-cause and in-home deaths and greater decreases in hospitalization among patients with cancer than among those without cancer. These changes were more prominent in the <85-year group (eFigure 1, eTable 3, and eTable 4).

In sex-stratified analyses, initiation of home-visit care was more common in male patients with cancer and female patients without cancer throughout both periods, although an increase was observed in both sexes during the pandemic. For outcomes, both sexes showed patterns consistent with the main analysis; however, the differences between patients with and without cancer in increases in all-cause and in-home deaths within 90 days were more pronounced in female patients than in male patients (eFigure 2, eTable 5, and eTable 6).

Regional analyses showed that initiation of home-visit care increased for both patients with and without cancer regardless of whether their prefecture was under the state of emergency designation. Similar to the main results, increases in all-cause death, in-home death, and emergency home visits, along with a decrease in hospitalizations, were more prominent among patients with cancer across all regions (eFigure 3, eTable 7, and eTable 8).

In cancer-type-specific analyses, prostate and lung cancers were the most common cancer types among male patients, whereas colorectal and lung cancers were the most common among female patients. Across cancer types, the number of newly initiated home-visit care patients increased during the pandemic, accompanied by a consistent pattern of reduced hospitalizations and increased all-cause and in-home deaths within 90 days after home-visit care initiation. Larger increases in in-home deaths were particularly evident for cancer types associated with higher 90-day mortality—such as pancreatic, lung, and gastric cancers—in both male and female patients (eFigure 4, eTable 9, and eTable 10).

## DISCUSSION

Using nationwide data, this study investigated the utilization and outcomes of the newly initiated physician regular home-visit care during the COVID-19 pandemic, with a particular focus on differences in patients with and without cancer. Our findings indicated that the number of patients initiating physician regular home visits increased during the COVID-19 pandemic in both cancer and non-cancer groups. The proportion of hospitalizations within 90 days decreased, whereas both all-cause and in-home deaths increased, with more pronounced changes observed among patients with cancer than among those without cancer. Although the number of telephone follow-up consultations increased in both groups, an increase in emergency home visits was observed only in patients with cancer.

This study revealed that the number of patients initiating physician regular home-visit care increased after the onset of the COVID-19 pandemic in both patients with and without cancer. However, the outcomes diverged significantly by group: compared with patients without cancer, those with cancer experienced a greater reduction in hospitalizations and a concomitant increase in home-based end-of-life care within 90 days after initiating home care. Three factors could explain this cancer-specific pattern. First, palliative care unit beds were curtailed during the pandemic, making home care a practical alternative for terminal cancer management.^16^ Second, since cancer confers a higher risk of severe COVID-19,^17^^,^^18^ some patients may have deliberately avoided hospitalization for fear of infection. Third, the relatively predictable clinical course of cancer allows patients and their families to plan for—and intentionally choose—death at home, particularly when hospital visits are restricted. Supporting this interpretation, a previous survey of home-visit physicians identified “restrictions on hospital visitation” as the primary driver (93.5%) of the increased demand for home care service.^19^

Notably, although a previous study reported that age-adjusted cancer mortality continued to decline during the pandemic,^20^ our study found that the proportion of patients who died within 90 days after initiating home-visit care increased. This suggests that more patients with limited life expectancy began using home-visit care during the pandemic, reflecting a shift toward home-based end-of-life care, even among those who might previously have remained hospitalized. Cancer-type–specific analyses further supported this pattern, showing particularly large increases in in-home deaths among patients with cancer types with high short-term mortality—such as pancreatic, lung, and gastric cancers—indicating that patients with the shorter life expectancy might have increasingly opted for end-of-life care at home during the pandemic.

A previous study using NDB open data^21^ reported no significant change in the monthly number of regular home-visit care claims during the COVID-19 pandemic. In contrast, our study showed that more patients began home-visit care during the pandemic. Although the total number of patients appeared stable each month, the increase in deaths within 90 days after starting care suggests a high turnover, which means that more patients started care and passed away in a short period. This likely places a substantial burden on physicians who provide home-based care. To ensure resilient care delivery, enhanced support through strengthened multidisciplinary collaboration is needed to assist both patients and their families. In addition, although telephone follow-up increased during the pandemic, use of telemedicine among patients receiving physician home-visit care remained limited.^22^ Expanding video-based telemedicine models with nurse support may help reduce the burden of frequent in-person visits on home-visit physicians.

During the COVID-19 pandemic, the use of telemedicine expanded globally.^23^ In Japan, regulations were also relaxed to allow telephone follow-up consultations for all patients.^24^ Reflecting these changes, our findings showed a marked increase in telephone follow-up consultations among patients receiving physician home-visit care, regardless of cancer diagnosis, with a slight but statistically significant increase observed among patients with cancer. In contrast, an increase in emergency home visits was observed only among patients with cancer. The slight decrease in emergency home visits among patients without cancer may be attributable to a greater reliance on telephone follow-up consultations. However, for patients with cancer—particularly those receiving end-of-life care—in-person visits may be necessary to directly address a wide range of symptoms.

In subgroup analyses, the increase in newly initiated home-visit care and in the proportions of all-cause and in-home deaths within 90 days was more pronounced in patients with cancer aged <85 years than in those aged ≥85 years. Similarly, female patients showed greater increases in all-cause and in-home deaths among patients with cancer compared with male patients. These findings align with previous reports indicating that, during the pandemic, home deaths increased particularly among younger and female patients with cancer.^9^ One possible explanation is that younger patients and their families may have increasingly preferred home-based end-of-life care to spend meaningful time together, rather than remaining hospitalized with restricted visitation. The greater increase in in-home deaths among female patients also aligns with findings from other countries.^5^ As female patients are more likely than male patients to express and discuss their preferred place of death,^25^ such increased engagement in end-of-life decision-making during the pandemic may have contributed to the rise in in-home deaths among female patients.

In regional analyses, increases in newly initiated home-visit care and in-home deaths during the pandemic—particularly among patients with cancer—were observed nationwide, regardless of whether a prefecture was under a state of emergency declaration. However, previous evidence indicates that rural areas in Japan rely more heavily on home-visit clinics with fewer physicians,^26^ suggesting that the per-physician care burden may have been greater in these regions. Therefore, although the overall trends did not differ substantially by region, rural home-visit providers may have faced disproportionate strain during the pandemic. To address these challenges, particularly in rural areas, clinicians should reinforce multidisciplinary collaboration with home-visiting nurses, pharmacists, and care managers to maintain continuity of care while preventing excessive burden on individual physicians. Implementing telemedicine models with nurse support could further reduce the need for frequent in-person visits. For administrators and policymakers, expanding training opportunities to increase the number of physicians able to provide home-based palliative and end-of-life care, securing flexible staffing support, and strengthening regional coordination to redistribute workload may be necessary to ensure timely access to home-based end-of-life care during future public health emergencies. At the same time, given that increases in home-visit care and in-home deaths during the pandemic—especially among patients with cancer—may have been influenced by hospital visitation restrictions and limited bed capacity, future pandemic preparedness should include systems that enable continuous hospital-based care, including online visitation and remote communication with families, as well as ensuring adequate hospital bed capacity.

This study has several limitations. First, the reasons for initiating home-visit care were not available, making it unclear whether home-based end-of-life care reflected the true preferences of patients and their families. Second, emergency home visits may have included visits for the purpose of death certification, and the data did not allow us to differentiate these from other types of visits. In addition, telephone follow-up consultations included both audio-only calls and video-based online consultations, which could not be distinguished in the data. Third, although differences in clinical severity may have affected home-visit care utilization and outcomes, we were unable to assess severity, such as cancer staging or performance status, because these data are not available in claims records. Although treatment information, such as surgery or chemotherapy, can be collected, it does not necessarily reflect clinical severity due to variations in the timing of diagnosis and in treatment intent. Fourth, our data were limited to the period up to March 2021, capturing only the early phase of the pandemic. Future research is needed to evaluate how the number and outcomes in newly initiated home visit patients have changed after the pandemic. Continued monitoring is also warranted to understand the long-term trends in the use of telephone follow-up consultations, which showed a marked increase during the pandemic.

In conclusion, in this national-level study in Japan, our study identified a significant increase in the initiation of home-visit care during the COVID-19 pandemic, along with a higher proportion of patients receiving end-of-life care within a short duration, especially among patients with cancer. These findings highlight the need to recognize that, during extraordinary situations, such as a pandemic, patients and their families may alter their preferences regarding end-of-life care settings. To ensure the resilience of home-visit care during any possible future public health emergencies, strengthening multidisciplinary collaboration and expanding the use of telemedicine models will be important. Policymakers could also consider strategic resource allocation, enhancing training opportunities for end-of-life care at home, securing flexible staffing support, and improving regional coordination to support timely access to home-based end-of-life care for patients with cancer.

## SUPPLEMENTARY MATERIAL

eMaterial 1. List of medical diagnosis categories and International Classification of Diseases 10th Revision codes

eTable 1. 90-day outcomes by pandemic period and cancer diagnosis, with April 2020 redefining the pandemic onset

eTable 2. Results of linear regression analyses examining changes in 90-day outcomes before and during the COVID-19 pandemic among patients with and without cancer, with April 2020 redefining the pandemic onset

eTable 3. 90-day outcomes by pandemic period and cancer diagnosis, stratified by age group

eTable 4. Results of linear regression analyses examining changes in 90-day outcomes before and during the COVID-19 pandemic among patients with and without cancer, stratified by age group

eTable 5. 90-day outcomes by pandemic period and cancer diagnosis, stratified by sex

eTable 6. Results of linear regression analyses examining changes in 90-day outcomes before and during the COVID-19 pandemic among patients with and without cancer, stratified by sex

eTable 7. 90-day outcomes by pandemic period and cancer diagnosis, stratified by the prefectures where a state of emergency was declared or not

eTable 8. Results of linear regression analyses examining changes in 90-day outcomes before and during the COVID-19 pandemic among patients with and without cancer, stratified by the prefectures where a state of emergency was declared or not

eTable 9. 90-day outcomes by pandemic period and cancer type among male patients

eTable 10. 90-day outcomes by pandemic period and cancer type among female patients

eFigure 1. Monthly trends in newly initiated physician home-visit care among patients with and without cancer, stratified by age group

eFigure 2. Monthly trends in newly initiated physician home-visit care among patients with and without cancer, stratified by age group

eFigure 3. Monthly trends in newly initiated physician home-visit care among patients with and without cancer, stratified by the prefectures where a state of emergency was declared or not

eFigure 4. Monthly trends in newly initiated physician home-visit care by cancer type and sex
