# Supplementary material for: Changes in Home-visit Care Utilization and Outcomes During the Coronavirus Disease 2019 Pandemic: Comparison Between Patients With and Without Cancer
Source: J Epidemiol. 2026 Oct 5;36(10):313–20. doi: 10.2188/jea.JE20250543 (PMC13562597; doi:10.2188/jea.JE20250543)
Supplement: Supplementary file 1 [file je-36-313-s001.pdf]

eMaterial 1. List of medical diagnosis categories and International Classification of Diseases 10th Revision codes

Medical diagnosis category	ICD-10 codes
1. Cerebrovascular diseases:	
- Hemorrhagic stroke	I60–I62
- Ischemic stroke	I63
- Other cerebrovascular diseases*	I64–I69
2. Cardiac diseases:	
- Ischemic heart disease	I20–I25
- Arrhythmia	I44, I45, I47–I49
- Heart failure	I50
- Other cardiac diseases	I01, I05–I09, I11, I13, I30–I43, I51, I52
3. Cancer	C00–C097
4. Lower respiratory tract diseases	
- Chronic obstructive pulmonary disease	J43, J44
- Pneumonia	J12–18, J69
- Other lower respiratory tract diseases	A15, A16, J20–J22, J40–J42, J45–J47, J60–J68, J70, J80–J86, J90–J94
5. Joint diseases	
- Rheumatoid arthritis	M05, M06
- Other arthropathies	M00–03, M07, M10–M25
- Dorsopathies (disorders of the back or spine)	M40–M54
6. Dementia	F00–F03, G30
7. Fractures	
- Femur fractures	S72
- Other fractures	S02, S12, S22, S32, S42, S52, S62, S82, S92, T02, T08, T10, T12

ICD-10, International Classification of Diseases 10th Revision.

*Including unspecified strokes and sequelae of cerebrovascular disease

eTable 1. 90-day outcomes by pandemic period and cancer diagnosis, with April 2020 redefining the pandemic onset

	Pre-pandemic period*			Pandemic period*		
	Total patients	Patients with cancer	Patients without cancer	Total patients	Patients with cancer	Patients without cancer
	n=342,819	n=166,884	n=175,935	n=166,899	n=84,427	n=82,472
	(%)	n (%)	n (%)	n (%)	n (%)	n (%)
(i) All-cause deaths	94,539 (27.6)	74,789 (44.8)	19,750 (11.2)	50,115 (30.0)	40,446 (47.9)	9,669 (11.7)
(ii) In-home deaths	58,443 (17.0)	46,358 (27.8)	12,085 (6.9)	34,450 (20.6)	28,082 (33.3)	6,368 (7.7)
(iii) At least one hospitalization	97,800 (28.5)	61,640 (36.9)	36,340 (20.7)	54,452 (32.6)	34,480 (32.3)	19,972 (18.9)
(iv) At least one emergency home visit	130,448 (38.1)	81,061 (48.6)	49,387 (28.1)	65,863 (39.5)	43,424 (51.4)	22,439 (27.2)
(v) At least one telephone follow-up consultation	47,475 (13.8)	27,067 (16.2)	20,408 (11.6)	33,445 (20.0)	21,158 (20.2)	12,287 (14.9)

*The pre-pandemic period included patients who newly initiated physician regular home visit care between April 2018 and March 2020, while the pandemic period included those who initiated care between April 2020 and December 2020.

eTable 2. Results of linear regression analyses examining changes in 90-day outcomes before and during the COVID-19 pandemic among patients with and without cancer, with April 2020 redefining the pandemic onset

	Cancer diagnosis					
	Patients with cancer		Patients without cancer			
	Adjusted difference (95% CI), percentage points	*P* value	Adjusted difference (95% CI), percentage points	*P* value	Relative difference (95% CI), percentage points	*P* value
(i) All-cause deaths	2.8 (2.1–3.6)	<0.001	0.5 (0.2–0.9)	0.001	2.3 (1.6–2.9)	<0.001
(ii) In-home deaths	5.3 (4.6–5.9)	<0.001	0.9 (0.6–1.1)	<0.001	4.4 (3.9–5.0)	<0.001
(iii) At least one hospitalization	-4.7 (-5.5 to -3.9)	<0.001	-1.6 (-2.0 to -1.2)	<0.001	-3.1 (-3.8 to -2.4)	<0.001
(iv) At least one emergency home visit	2.7 (2.0–3.4)	<0.001	-0.8 (-1.2 to -0.4)	0.001	3.5 (2.9–4.1)	<0.001
(v) At least one telephone follow-up consultation	4.0 (3.4–4.6)	<0.001	3.3 (2.6–3.9)	<0.001	0.8 (0.3–1.2)	0.002

CI, confidence interval; COVID-19, coronavirus disease 2019.

Linear regression analyses were performed by regressing each outcome on three variables: (1) a cancer indicator, (2) a pandemic-period indicator, and (3) their interaction. Models were adjusted for prefecture fixed effects and patient covariates (age category, sex, and medical diagnosis at the start of regular home visits) to account for regional heterogeneity and case-mix. Standard errors were clustered at the prefecture level to account for correlations among patients within the same prefecture.

eFigure 1. Monthly trends in newly initiated physician home-visit care among patients with and without cancer, stratified by age group

A. <85 years old   B. ≥85 years old

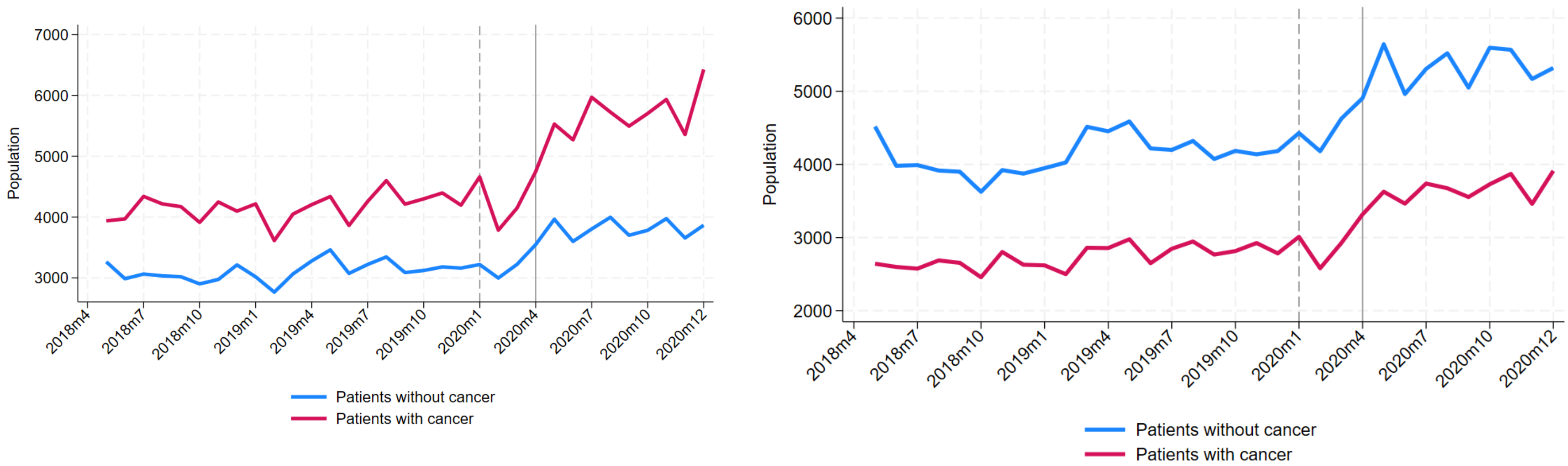

The gray dashed line indicates the month of the first confirmed COVID-19 case in Japan and marks the boundary between the pre-pandemic and during-pandemic periods defined in this study. The gray solid line represents the month in which the state of emergency was declared in Japan. (**A**) Age <85 years; (**B**) Age ≥85 years.

eTable 3. 90-day outcomes by pandemic period and cancer diagnosis, stratified by age group

	Pre-pandemic period*			Pandemic period*		
	Total patients	Patients with cancer	Patients without cancer	Total patients	Patients with cancer	Patients without cancer
<85 years old	n=153,245, n (%)	n=87,788, n (%)	n=65,457, n (%)	n=108,187, n (%)	n=64,078, n (%)	n=44,109, n (%)
(i) All-deaths	50,037 (32.7)	44,589 (50.8)	5,448 (8.3)	38,063 (35.2)	34,218 (53.4)	3,845 (8.7)
(ii) In-home deaths	29,905 (19.5)	27,117 (30.9)	2,788 (4.3)	25,697 (23.8)	23,514 (36.7)	2,183 (4.9)
(iii) At least one hospitalization	50,541 (33.0)	36,003 (41.0)	14,538 (22.2)	31,635 (29.2)	22,718 (35.5)	8,917 (20.2)
(iv) At least one emergency home visit	62,344 (40.7)	45,216 (51.5)	17,128 (26.2)	45,915 (42.4)	34,801 (54.3)	11,114 (25.2)
(v) At least one telephone follow-up consultation	22,783 (14.9)	14,855 (16.9)	7,928 (12.1)	20,710 (19.1)	13,634 (21.3)	7,076 (16.0)
≥85 years old	n=144,598, n (%)	n=57,598, n (%)	n=87,000, n (%)	n=103,688, n (%)	n=41,847, n (%)	n=61,841, n (%)
(i) All-deaths	32,277 (22.3)	20,658 (35.9)	11619 (13.4)	24,277 (23.4)	15,770 (37.7)	8,507 (13.8)
(ii) In-home deaths	20,447 (14.1)	12,921 (22.4)	7526 (8.7)	16,844 (16.2)	10,888 (26.0)	5,956 (9.6)
(iii) At least one hospitalization	35,836 (24.8)	18,425 (32.0)	17411 (20.0)	22,817 (22.0)	11,762 (28.1)	11,055 (17.9)
(iv) At least one emergency home visit	51,151 (35.4)	25,418 (44.1)	25733 (29.6)	36,901 (35.6)	19,050 (45.5)	17,851 (28.9)
(v) At least one telephone follow-up consultation	17,162 (11.9)	8,131 (14.1)	9031 (10.4)	16,179 (15.6)	7,524 (18.0)	8,655 (14.0)

*The pre-pandemic period included patients who newly initiated physician regular home visit care between April 2018 and December 2019, while the pandemic period included those who initiated care between January 2020 and December 2020.

eTable 4. Results of linear regression analyses examining changes in 90-day outcomes before and during the COVID-19 pandemic among patients with and without cancer, stratified by age group

	Cancer diagnosis					
	Patients with cancer		Patients without cancer			
	Adjusted difference (95% CI), percentage points	*P* value	Adjusted difference (95% CI), percentage points	*P* value	Relative difference (95% CI), percentage points	*P* value
<85 years old						
(i) All-cause deaths	2.4 (1.7–3.1)	<0.001	0.3 (-0.1 to 0.6)	0.097	2.1 (1.4–2.7)	<0.001
(ii) In-home deaths	5.7 (5.0–6.3)	<0.001	0.6 (0.3–0.9)	<0.001	5.1 (4.5–5.7)	<0.001
(iii) At least one hospitalization	-5.6 (-6.6 to -4.7)	<0.001	-1.9 (-2.4 to -1.4)	<0.001	-3.8 (-4.7 to -2.8)	<0.001
(iv) At least one emergency home visit	2.7 (1.8–3.5)	<0.001	-1.0 (-1.5 to -0.5)	<0.001	3.7 (2.7–4.6)	<0.001
(v) At least one telephone follow-up consultation	4.3 (3.6–5.1)	<0.001	3.8 (3.2–4.4)	<0.001	0.5 (-0.1 to 1.1)	0.103
≥85 years old						
(i) All-cause deaths	1.8 (1.0–2.6)	<0.001	0.6 (0.1–1.0)	0.009	1.3 (0.1–1.9)	<0.001
(ii) In-home deaths	3.6 (2.9–4.2)	<0.001	1.1 (0.7–1.5)	<0.001	2.5 (2.0–2.9)	<0.001
(iii) At least one hospitalization	-3.7 (-4.6 to -2.9)	<0.001	-2.1 (-2.6 to -1.6)	<0.001	-1.7 (-2.5 to -0.8)	<0.001
(iv) At least one emergency home visit	1.4 (0.7–2.2)	<0.001	-0.6 (-1.2 to 0.0)	0.044	2.0 (1.4–2.6)	<0.001
(v) At least one telephone follow-up consultation	3.9 (3.3–4.5)	<0.001	3.5 (2.9–4.2)	<0.001	0.3 (-0.1 to 0.8)	0.131

CI, confidence interval; COVID-19, coronavirus disease 2019.

Linear regression analyses were performed by regressing each outcome on three variables: (1) a cancer indicator, (2) a pandemic-period indicator, and (3) their interaction. Models were adjusted for prefecture fixed effects and patient covariates (age category, sex, and medical diagnosis at the start of regular home visits) to account for regional heterogeneity and case-mix. Standard errors were clustered at the prefecture level to account for correlations among patients within the same prefecture.

eFigure 2. Monthly trends in newly initiated physician home-visit care among patients with and without cancer, stratified by age group

A. Male

B. Female

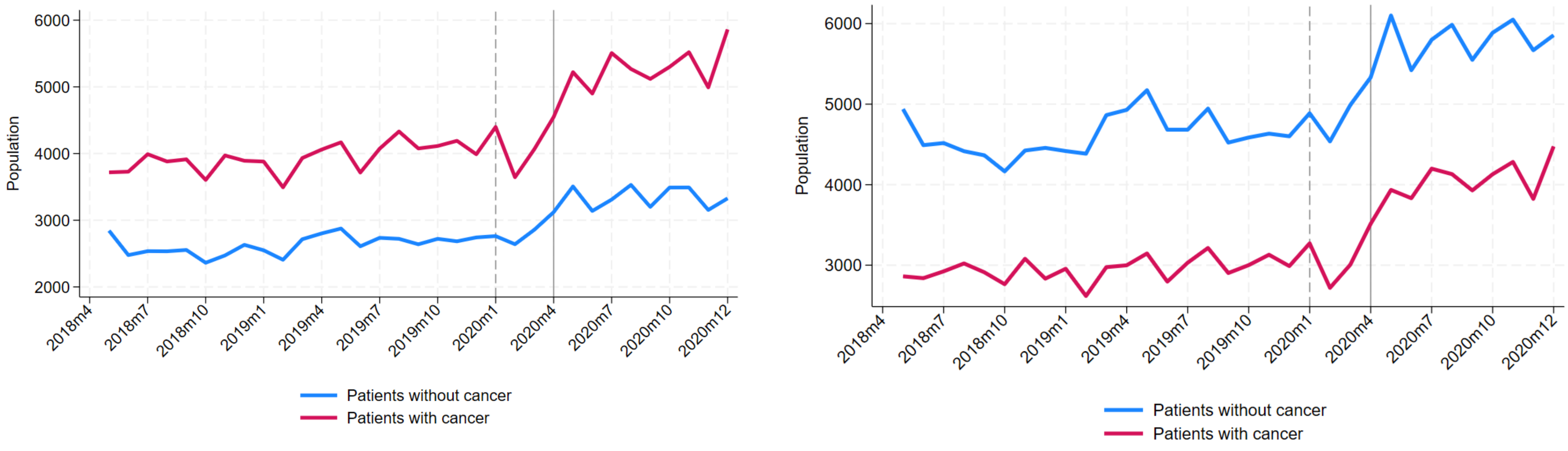

The gray dashed line indicates the month of the first confirmed COVID-19 case in Japan and marks the boundary between the pre-pandemic and during-pandemic periods defined in this study. The gray solid line represents the month in which the state of emergency was declared in Japan. (**A**) male; (**B**) female.

eTable 5. 90-day outcomes by pandemic period and cancer diagnosis, stratified by sex

	Pre-pandemic period*			Pandemic period*		
	Total patients	Patients with cancer	Patients without cancer	Total patients	Patients with cancer	Patients without cancer
Male	n=138,492, n (%)	n=83,120, n (%)	n=55,372, n (%)	n=98,712, n (%)	n=59,946, n (%)	n=38,766, n (%)
(i) All-deaths	47,405 (34.2)	39,820 (47.9)	7,585 (13.7)	35,466 (35.9)	29,935 (49.9)	5,531 (14.3)
(ii) In-home deaths	28,380 (20.5)	24,241 (29.2)	4,139 (7.5)	23,735 (24.0)	20,341 (33.9)	3,394 (8.8)
(iii) At least one hospitalization	46,006 (33.2)	32,290 (38.8)	13,716 (24.8)	28,775 (29.2)	20,095 (33.5)	8,680 (22.4)
(iv) At least one emergency home visit	58,567 (42.3)	41,826 (50.3)	16,741 (30.2)	42,884 (43.4)	31,421 (52.4)	11,463 (29.6)
(v) At least one telephone follow-up consultation	19,602 (14.2)	13,141 (15.8)	6,461 (11.7)	18,019 (18.3)	11,993 (20.0)	6,026 (15.5)
Female	n=159,351, n (%)	n=62,266, n (%)	n=97,085, n (%)	n=113,163, n (%)	n=45,979, n (%)	n=67,184, n (%)
(i) All-deaths	34,909 (21.9)	25,427 (40.8)	9,482 (9.8)	26,874 (23.7)	20,053 (43.6)	6,821 (10.2)
(ii) In-home deaths	21,972 (13.8)	15,797 (25.4)	6,175 (6.4)	18,806 (16.6)	14,061 (30.6)	4,745 (7.1)
(iii) At least one hospitalization	40,371 (25.3)	22,138 (35.6)	18,233 (18.8)	25,677 (22.7)	14,385 (31.3)	11,292 (16.8)
(iv) At least one emergency home visit	54,928 (34.5)	28,808 (46.3)	26,120 (26.9)	39,932 (35.3)	22,430 (48.8)	17,502 (26.1)
(v) At least one telephone follow-up consultation	20,343 (12.8)	9,845 (15.8)	10,498 (10.8)	18,870 (16.7)	9,165 (19.9)	9,705 (14.4)

*The pre-pandemic period included patients who newly initiated physician regular home visit care between April 2018 and December 2019, while the pandemic period included those who initiated care between January 2020 and December 2020.

eTable 6. Results of linear regression analyses examining changes in 90-day outcomes before and during the COVID-19 pandemic among patients with and without cancer, stratified by sex

	Cancer diagnosis					
	Patients with cancer		Patients without cancer			
	Adjusted difference (95% CI), percentage points	*P* value	Adjusted difference (95% CI), percentage points	*P* value	Relative difference (95% CI), percentage points	*P* value
Male						
(i) All-cause deaths	1.8 (1.0–2.5)	<0.001	0.6 (0.8–1.1)	0.025	1.2 (0.5–1.9)	0.001
(ii) In-home deaths	4.6 (3.9–5.3)	<0.001	1.3 (0.9–1.6)	<0.001	3.3 (2.7–4.0)	<0.001
(iii) At least one hospitalization	-5.3 (-6.4 to -4.3)	<0.001	-2.2 (-2.7 to -1.7)	<0.001	-3.2 (-4.4 to -1.9)	<0.001
(iv) At least one emergency home visit	1.9 (1.0–2.8)	<0.001	-0.6 (-1.5 to 0.2)	0.148	2.6 (1.7–3.5)	<0.001
(v) At least one telephone follow-up consultation	4.2 (3.6–4.8)	<0.001	3.9 (3.1–4.6)	<0.001	0.4 (-0.4 to 1.2)	0.363
Female						
(i) All-cause deaths	2.6 (1.8–3.4)	<0.001	0.5 (0.1–0.9)	0.008	2.1 (1.4–2.7)	<0.001
(ii) In-home deaths	5.1 (4.6–5.6)	<0.001	0.8 (0.5–1.1)	<0.001	4.3 (3.8–4.8)	<0.001
(iii) At least one hospitalization	-4.3 (-5.1 to -3.4)	<0.001	-1.8 (-2.4 to -1.2)	<0.001	-2.5 (-3.2 to -1.8)	<0.001
(iv) At least one emergency home visit	2.4 (1.7–3.1)	<0.001	-0.7 (-1.2 to 0.3)	0.002	3.1 (2.3–4.0)	<0.001
(v) At least one telephone follow-up consultation	4.1 (3.1–5.0)	<0.001	3.6 (3.0–4.1)	<0.001	0.5 (-0.1 to 1.1)	0.108

CI, confidence interval; COVID-19, coronavirus disease 2019.

Linear regression analyses were performed by regressing each outcome on three variables: (1) a cancer indicator, (2) a pandemic-period indicator, and (3) their interaction. Models were adjusted for prefecture fixed effects and patient covariates (age category, sex, and medical diagnosis at the start of regular home visits) to account for regional heterogeneity and case-mix. Standard errors were clustered at the prefecture level to account for correlations among patients within the same prefecture.

eFigure 3. Monthly trends in newly initiated physician home-visit care among patients with and without cancer, stratified by the prefectures where a state of emergency was declared or not

A. Prefectures where a state of emergency was declared

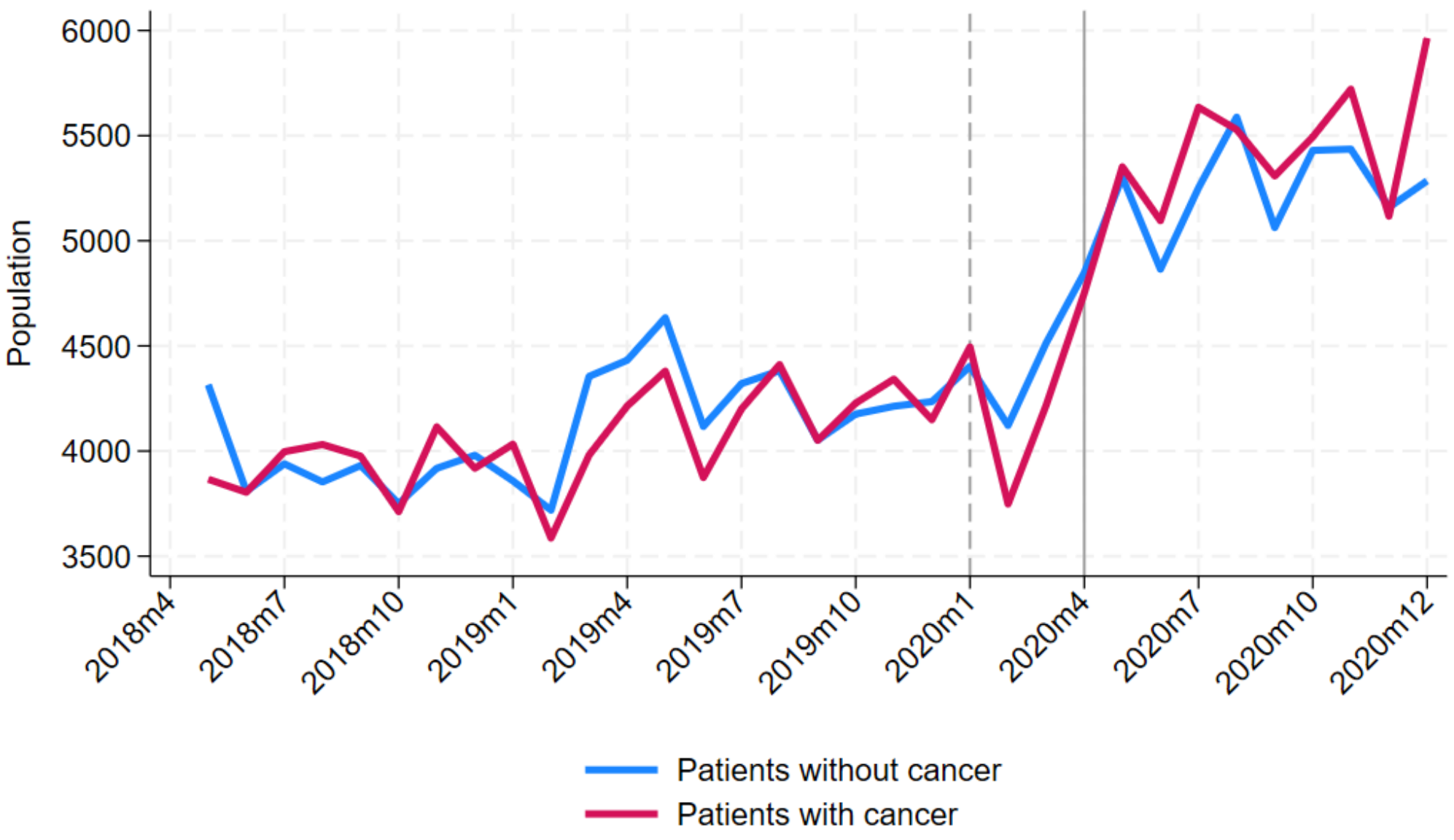

B. Prefectures where a state of emergency was not declared

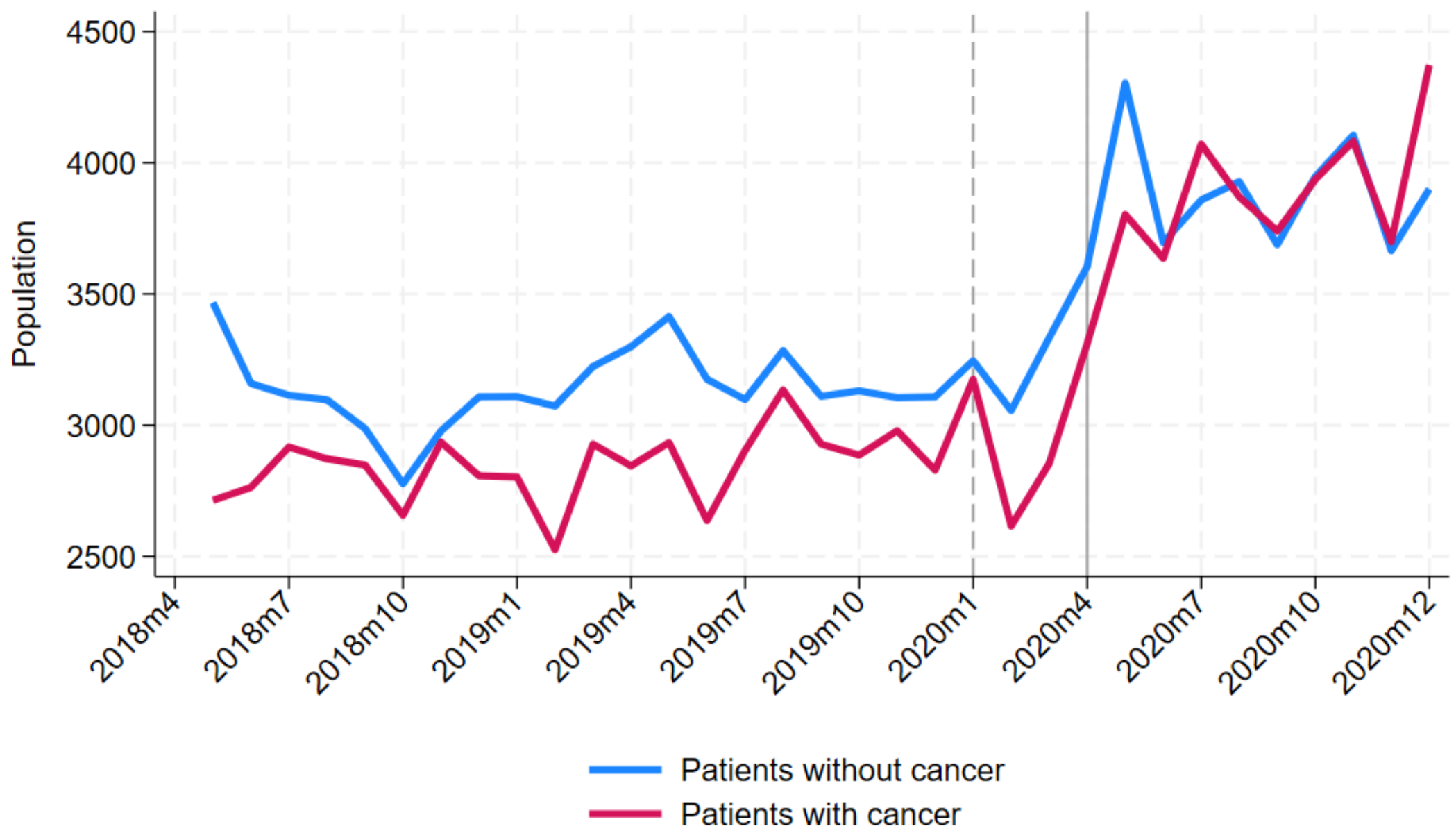

The gray dashed line indicates the month of the first confirmed COVID-19 case in Japan and marks the boundary between the pre-pandemic and during-pandemic periods defined in this study. The gray solid line represents the month in which the state of emergency was declared in Japan. (**A**) Prefectures where a state of emergency was declared; (**B**) Prefectures where a state of emergency was not declared.

eTable 7. 90-day outcomes by pandemic period and cancer diagnosis, stratified by the prefectures where a state of emergency was declared or not

	Pre-pandemic period*			Pandemic period*		
	Total patients	Patients with cancer	Patients without cancer	Total patients	Patients with cancer	Patients without cancer
Prefectures where a state of emergency was declared	n=171,756, n (%)	n=85,363, n (%)	n=86,393, n (%)	n=122,799, n (%)	n=61,934, n (%)	n=60,865, n (%)
(i) All-deaths	45,753 (26.6)	37,085 (43.4)	8,668 (10.0)	34,277 (27.9)	27,988 (45.2)	6,289 (10.3)
(ii) In-home deaths	27,188 (15.8)	22,113 (25.9)	5,075 (5.9)	22,931 (18.7)	18,899 (30.5)	4,032 (6.6)
(iii) At least one hospitalization	50,013 (29.1)	32,051 (37.5)	17,962 (20.8)	31,287 (25.5)	20,063 (32.4)	11,224 (18.4)
(iv) At least one emergency home visit	65,480 (38.1)	40,932 (48.0)	24,548 (28.4)	47,551 (38.7)	30,891 (49.9)	16,660 (27.4)
(v) At least one telephone follow-up consultation	27,845 (16.2)	15,960 (18.7)	11,885 (13.8)	25,310 (20.6)	14,441 (23.3)	10,869 (17.9)
Prefectures where a state of emergency was not declared	n=126,087, n (%)	n=60,023, n (%)	n=66,064, n (%)	n=89,076, n (%)	n=43,991, n (%)	n=45,085, n (%)
(i) All-deaths	36,561 (29.0)	28,162 (46.9)	8,399 (12.7)	28,063 (31.5)	22,000 (50.0)	6,063 (13.5)
(ii) In-home deaths	23,164 (18.4)	17,925 (27.6)	5,239 (6.9)	19,610 (22.0)	15,503 (32.8)	4,107 (8.0)
(iii) At least one hospitalization	36,364 (28.8)	22,377 (37.3)	13,987 (21.2)	23,165 (26.0)	14,417 (32.8)	8,748 (19.4)
(iv) At least one emergency home visit	48,015 (38.1)	29,702 (49.5)	18,313 (27.7)	35,265 (39.6)	22,960 (52.2)	12,305 (27.3)
(v) At least one telephone follow-up consultation	12,100 (9.6)	7,026 (11.7)	5,074 (7.7)	11,579 (13.0)	6,717 (15.3)	4,862 (10.8)

*The pre-pandemic period included patients who newly initiated physician regular home visit care between April 2018 and December 2019, while the pandemic period included those who initiated care between January 2020 and December 2020.

eTable 8. Results of linear regression analyses examining changes in 90-day outcomes before and during the COVID-19 pandemic among patients with and without cancer, stratified by the prefectures where a state of emergency was declared or not

	Cancer diagnosis					
	Patients with cancer		Patients without cancer			
	Adjusted difference (95% CI), percentage points	*P* value	Adjusted difference (95% CI), percentage points	*P* value	Relative difference (95% CI), percentage points	*P* value
Prefectures where a state of emergency was declared						
(i) All-cause deaths	1.6 (0.5–2.8)	0.013	0.3 (-0.1 to 0.8)	0.086	1.3 (0.4–2.1)	0.010
(ii) In-home deaths	4.5 (3.6–5.5)	<0.001	0.8 (0.4–1.1)	0.002	3.7 (2.9–4.6)	<0.001
(iii) At least one hospitalization	-5.2 (-6.6 to -3.7)	<0.001	-2.3 (-2.8 to -1.7)	<0.001	-2.9 (-4.2 to -1.5)	0.002
(iv) At least one emergency home visit	1.9 (0.5–3.3)	0.017	-0.1 (-0.2 to 0.0)	0.019	2.9 (1.6–4.2)	0.002
(v) At least one telephone follow-up consultation	4.6 (3.7–5.5)	<0.001	4.1 (3.3–5.0)	<0.001	0.5 (-0.1 to 1.1)	0.103
Prefectures where a state of emergency was not declared						
(i) All-cause deaths	2.8 (2.1–3.5)	<0.001	0.8 (0.3–1.3)	0.003	2.0 (1.4–2.6)	<0.001
(ii) In-home deaths	5.2 (4.5–5.8)	<0.001	1.1 (0.7–1.6)	<0.001	4.0 (3.3–4.7)	<0.001
(iii) At least one hospitalization	-4.5 (-5.7 to -3.4)	<0.001	-1.5 (-2.0 to -1.1)	<0.001	-3.0 (-4.1 to -1.9)	<0.001
(iv) At least one emergency home visit	2.5 (1.7–3.3)	<0.001	-0.4 (-1.0 to 0.3)	0.254	2.8 (1.9–3.8)	<0.001
(v) At least one telephone follow-up consultation	3.5 (2.5–4.5)	<0.001	3.1 (2.4–3.7)	<0.001	0.4 (-0.4 to 1.3)	0.331

CI, confidence interval; COVID-19, coronavirus disease 2019.

Linear regression analyses were performed by regressing each outcome on three variables: (1) a cancer indicator, (2) a pandemic-period indicator, and (3) their interaction. Models were adjusted for prefecture fixed effects and patient covariates (age category, sex, and medical diagnosis at the start of regular home visits) to account for regional heterogeneity and case-mix. Standard errors were clustered at the prefecture level to account for correlations among patients within the same prefecture.

eFigure 4. Monthly trends in newly initiated physician home-visit care by cancer type and sex

A. Male

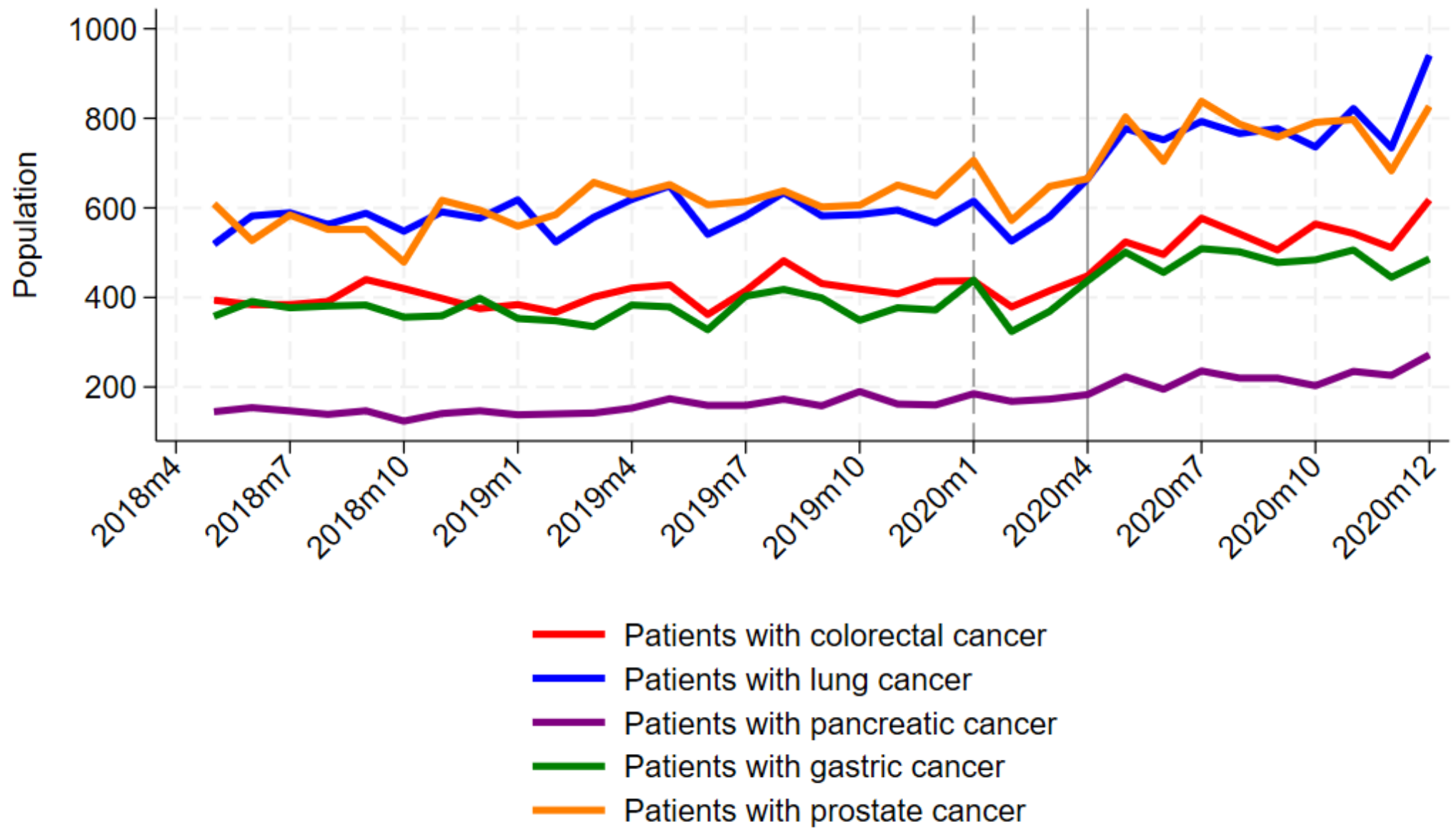

B. Female

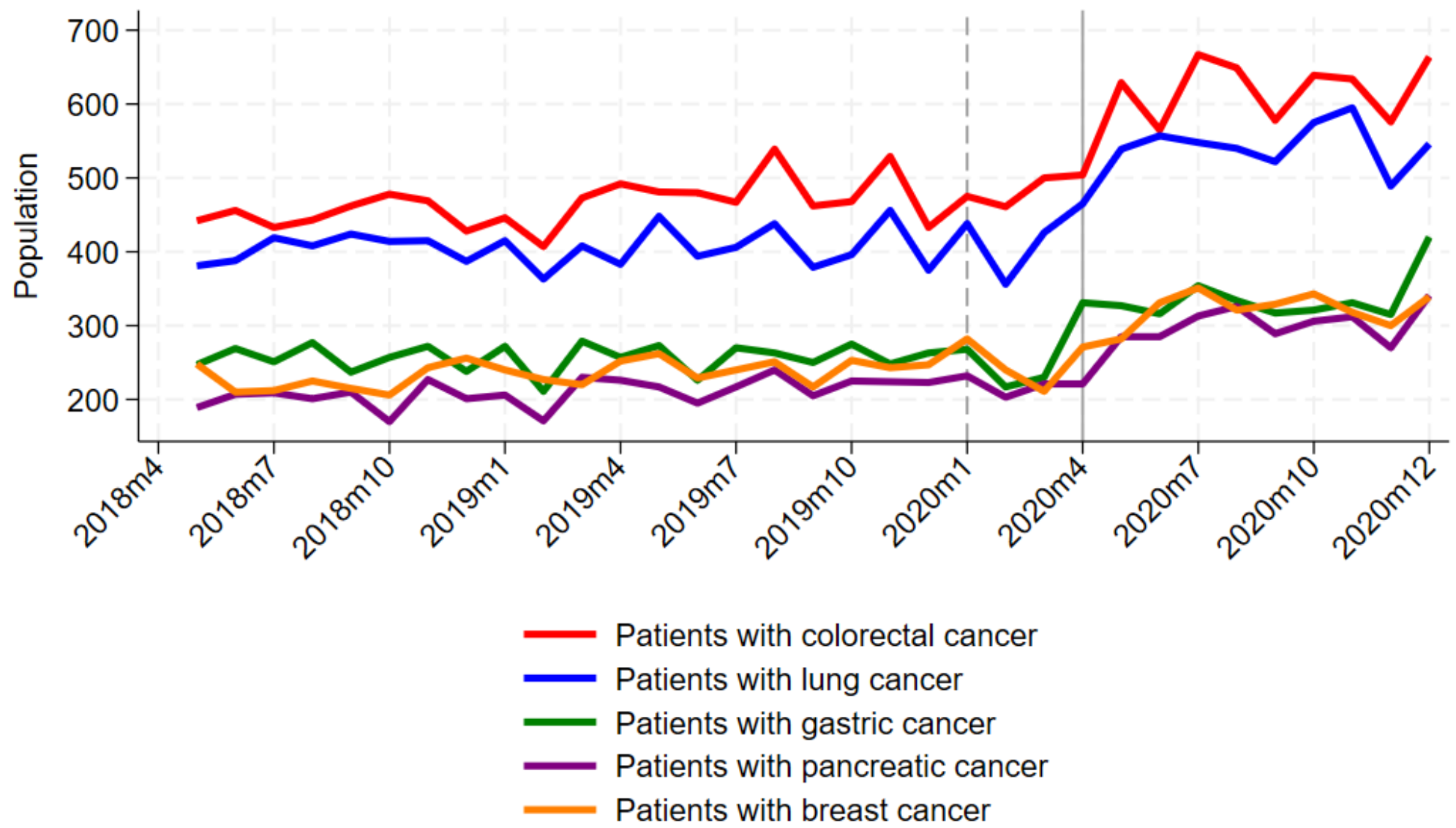

The gray dashed line indicates the month of the first confirmed COVID-19 case in Japan and marks the boundary between the pre-pandemic and during-pandemic periods defined in this study. The gray solid line represents the month in which the state of emergency was declared in Japan. (**A**) Male; (**B**) Female.

eTable 9. 90-day outcomes by pandemic period and cancer type among male patients

	Pre-pandemic period*					Pandemic period*				
	Colorectal cancer	Lung cancer	Gastric cancer	Pancreatic cancer	Prostate cancer	Colorectal cancer	Lung cancer	Gastric cancer	Pancreatic cancer	Prostate cancer
	n=8,577	n=12,246	n=7,886	n=3,237	n=12,648	n=6,123	n=8,869	n=5,497	n=2,554	n=8,873
	(%)	n (%)	(%)	n (%)	n (%)	n (%)	n (%)	(%)	n (%)	n (%)
(i) All-cause deaths	3,832 (44.7)	6,887 (56.2)	4,093 (51.9)	2,099 (64.8)	3,430 (27.1)	2,754 (45.0)	5,259 (59.3)	2,966 (54.0)	1,768 (69.2)	2,457 (27.7)
(ii) In-home deaths	2,351 (27.4)	4,016 (32.8)	2,610 (33.1)	1,378 (42.6)	2,091 (16.5)	1,866 (30.5)	3,476 (39.2)	2,076 (37.8)	1,271 (49.8)	1,636 (18.4)
(iii) At least one hospitalization	1,741 (20.3)	2,898 (23.7)	1,626 (20.6)	690 (21.3)	2,085 (16.5)	1,127 (18.4)	1,747 (19.7)	1,016 (18.5)	505 (19.8)	1,300 (14.7)
(iv) At least one emergency home visit	4,164 (48.5)	6,477 (52.9)	4,158 (52.7)	1,915 (59.2)	5,193 (41.1)	3,038 (49.6)	4,963 (56.0)	3,014 (54.8)	1,662 (65.1)	3,586 (40.4)
(v) At least one telephone follow-up consultation	1,289 (15.0)	1,943 (15.9)	1,184 (15.0)	564 (17.4)	1,957 (15.5)	1,162 (19.0)	1,727 (19.5)	1,045 (19.0)	537 (21.0)	1,715 (19.3)

*The pre-pandemic period included patients who newly initiated physician regular home visit care between April 2018 and December 2019, while the pandemic period included those who initiated care between January 2020 and December 2020.

eTable 10. 90-day outcomes by pandemic period and cancer type among female patients

	Pre-pandemic period*					Pandemic period*				
	Colorectal cancer	Lung cancer	Gastric cancer	Pancreatic cancer	Breast cancer	Colorectal cancer	Lung cancer	Gastric cancer	Pancreatic cancer	Breast cancer
	n=9,763	n=8,535	n=5,403	n=4,425	n=4,978	n=7,066	n=6,158	n=3,813	n=3,372	n=3,636
	(%)	n (%)	(%)	n (%)	n (%)	n (%)	n (%)	(%)	n (%)	n (%)
(i) All-cause deaths	3,371 (34.5)	3,485 (40.8)	2,182 (40.4)	2,474 (55.9)	1,770 (35.6)	2,641 (37.4)	2,720 (44.2)	1,611 (42.3)	1,953 (57.9)	1,388 (38.2)
(ii) In-home deaths	2,166 (22.2)	2,075 (24.3)	1,432 (26.5)	1,564 (35.3)	1,069 (21.5)	1,859 (26.3)	1,858 (30.2)	1,178 (30.9)	1,413 (41.9)	909 (25.0)
(iii) At least one hospitalization	1,789 (18.3)	1,822 (21.3)	1,012 (18.7)	958 (21.6)	900 (18.1)	1,175 (16.6)	1,191 (19.3)	661 (17.3)	634 (18.8)	618 (17.0)
(iv) At least one emergency home visit	4,137 (42.4)	3,819 (44.7)	2,521 (46.7)	2,430 (54.9)	2,119 (42.6)	3,177 (45.0)	2,983 (48.4)	1,846 (48.4)	1,918 (56.9)	1,577 (43.4)
(v) At least one telephone follow-up consultation	1,446 (14.8)	1,278 (15.0)	825 (15.3)	776 (17.5)	754 (15.1)	1,370 (19.4)	1,178 (19.1)	732 (19.2)	671 (19.9)	706 (19.4)

*The pre-pandemic period included patients who newly initiated physician regular home visit care between April 2018 and December 2019, while the pandemic period included those who initiated care between January 2020 and December 2020.
